# Associations between sedentary behaviours and dietary intakes among adolescents

**DOI:** 10.1017/S136898001700372X

**Published:** 2018-01-10

**Authors:** Elly A Fletcher, Sarah A McNaughton, David Crawford, Verity Cleland, Jacqueline Della Gatta, Jennifer Hatt, James Dollman, Anna Timperio

**Affiliations:** 1 Institute for Physical Activity and Nutrition (IPAN), School of Exercise and Nutrition Sciences, Deakin University, 221 Burwood Highway, Burwood, VIC 3125, Australia; 2 Menzies Institute for Medical Research, University of Tasmania, Hobart, Tasmania, Australia; 3 Alliance for Research in Exercise, Nutrition and Activity (ARENA), School of Health Sciences, University of South Australia, Adelaide, South Australia, Australia

**Keywords:** Television viewing, Screen time, Sitting, Diet, Snacks, Adolescents

## Abstract

**Objective:**

The purpose of the current study was to examine associations of individual and aggregated screen-based behaviours, and total sitting time, with healthy and unhealthy dietary intakes among adolescents.

**Design:**

Cross-sectional study of adolescents. Participants self-reported durations of television viewing, computer use, playing electronic games (e-games), total sitting time, daily servings of fruits and vegetables, and frequency of consumption of sugar-sweetened beverages (SSB), diet beverages, fast foods and discretionary snacks. Logistic regression models were conducted to identify associations of screen-based behaviours, total screen time and total sitting time with dietary intakes.

**Setting:**

Victoria, Australia.

**Subjects:**

Adolescents (*n* 939) in School Year 11 (mean age 16·8 years).

**Results:**

The results showed that watching television (≥2 h/d) was positively associated with consuming SSB and diet beverages each week and consuming discretionary snacks at least once daily, whereas computer use (≥2 h/d) was inversely associated with daily fruit and vegetable intake and positively associated with weekly fast-food consumption. Playing e-games (any) was inversely associated with daily vegetable intake and positively associated with weekly SSB consumption. Total screen (≥2 h/d) and sitting (h/d) times were inversely associated with daily fruit and vegetable consumption, with total screen time also positively associated with daily discretionary snack consumption and weekly consumption of SSB and fast foods.

**Conclusions:**

Individual and aggregated screen-based behaviours, as well as total sitting time, are associated with a number of indicators of healthy and unhealthy dietary intake. Future research should explore whether reducing recreational screen time improves adolescents’ diets.

Less than a third of adolescents in Western countries meet sedentary behaviour guidelines that recommend they participate in screen time for no more than 2 h/d^(^ ^1^ ^–^ ^3^ ^)^. This is concerning because evidence among adolescents suggests that high amounts of television (TV) viewing increase the risk of overweight and obesity and metabolic syndrome, independent of leisure-time physical activity^(^ ^4^ ^,^ ^5^ ^)^. There are several potential mechanisms for this. Among adults, there is some evidence that prolonged sitting may impair metabolic processes^(^ ^6^ ^)^. Another hypothesis is that behaviours such as TV viewing displace physical activity. However, a meta-analyses involving 163 studies found only a small inverse association between sedentary behaviour and physical activity, suggesting that these behaviours do not directly displace one another^(^ ^7^ ^)^. It has also been suggested that sedentary behaviour is associated with poor dietary habits, which may also help explain adverse health outcomes. Watching TV, for example, has been linked with consuming fewer fruits and vegetables, higher intakes of discretionary snacks, sugar-sweetened beverages (SSB) and takeaway foods, and greater overall energy intake^(^ ^8^ ^)^. Indeed, several studies have demonstrated a tendency for ‘unhealthy’ behaviours such as sedentary behaviour and diet to cluster together^(^ ^9^ ^)^.

Apart from TV viewing, few studies have examined associations of other sedentary screen-based behaviours, such as computer use and playing electronic games (e-games), and overall sedentary time with dietary intake^(^ ^10^ ^–^ ^14^ ^)^. Of the existing studies, the majority have examined an aggregate measure of screen-based sedentary behaviours (e.g. ‘total screen time’)^(^ ^10^ ^,^ ^11^ ^)^ or a combined measure of dietary intakes (e.g. combining all ‘unhealthy’ foods)^(^ ^12^ ^)^. To date, studies that have explored associations between a range of individual sedentary behaviours and dietary intake have focused on children^(^ ^13^ ^,^ ^14^ ^)^. Those studies found that TV viewing was associated with a number of unhealthy dietary intakes among 9–11-year-old children, such as higher consumption of soft drinks, sweets, pastries, fried foods and fast foods, and lower fruit and vegetable consumption^(^ ^13^ ^,^ ^14^ ^)^. Computer use was associated only with higher energy intake^(^ ^13^ ^)^, and overall sedentary time was associated only with the consumption of sport drinks^(^ ^14^ ^)^. However, little is known about whether these associations observed in children are evident among adolescents.

Adolescents in Western countries have notoriously poor diets, consuming more SSB and fast foods than any other age group^(^ ^15^ ^–^ ^17^ ^)^. In Australia, for example, a recent national dietary survey highlighted that only 5 % of adolescents consumed recommended quantities of fruits and vegetables, over 50 % of adolescents consumed SSB on the day of the survey, and energy-dense, nutrient-poor discretionary foods and beverages (such as confectionery, SSB, takeaway foods) contributed a total of 41 % of energy intake^(^ ^15^ ^)^. Poor diets are important contributors to the burden of disease in Australia^(^ ^18^ ^)^. Among youth, eating too few fruits and vegetables and consumption of SSB are associated with overweight and obesity and poorer cardiometabolic health^(^ ^19^ ^)^. Exploring the associations between various sedentary behaviours and a range of eating habits may help to inform future interventions to improve overall lifestyle behaviours in adolescents. The current study aimed to determine associations of individual and aggregated screen-based behaviours, and total sitting time, with healthy (e.g. fruit and vegetable intakes) and unhealthy (e.g. discretionary snacking, SSB consumption, and fast-food intakes) dietary intake among adolescents.

## Methods

### Study design

The present study draws on baseline cross-sectional data from the ProjectADAPT study; a 3-year longitudinal study that recruited participants in Year 11 (approximately 16–17 years old) and tracked their behaviours into young adulthood. Baseline data were collected between August 2013 and June 2015.

### Participant recruitment

Participants were recruited through secondary schools and online advertising. Schools with at least fifty students enrolled in Year 11 (second-last year of secondary school in Australia) were selected across strata (tertiles) of area-level socio-economic status (SES) within (i) urban and (ii) rural areas of Victoria. Area-level SES was based on the Index of Relative Socio-Economic Advantage and Disadvantage, compiled by the Australian Bureau of Statistics, which takes into account pockets of advantage within disadvantaged postcodes^(^ ^20^ ^)^. Urban *v*. rural areas were defined using the Australian Bureau of Statistics’ Remoteness Index (major city or regional/remote)^(^ ^21^ ^)^. Participating schools (*n* 47) distributed information about the study to Year 11 students from August 2013 to September 2014. Consent was received from 382 participants (4 % of recruitment packs distributed). As the response was low, an advertisement for the study was placed on an online social networking site (Facebook) to complement recruitment via schools. The advertisement was limited to participants aged 16–17 years living in Victoria, Australia and ran from September to November 2014 and from April to May 2015. The advertisement directed individuals to a study website where they could register their interest to receive detailed information about the study. Consent was received from 640 participants recruited through social media (of 2770 registrations of interest). In total, 1076 consent forms were received, and baseline surveys were completed by 1022 participants. In 2013, participants completed the survey via telephone. Participants recruited from 2014 could opt to complete surveys online or via telephone interview. In total, seventy-six participants completed the survey via telephone.

### Measures

#### Sedentary behaviour

Total time usually spent watching TV, using a computer, laptop or tablet for leisure, and playing e-games for leisure during the week (Monday to Friday) and the weekend (Saturday and Sunday) was self-reported (eight separate items); these items were adapted from an existing instrument^(^ ^22^ ^)^. Responses for weekdays and weekends were summed for each individual behaviour and converted to average hours per day. Each individual behaviour was summed to compute total screen time. As all individual sedentary behaviour variables were negatively skewed, watching TV, using a computer and total screen time were each dichotomized as <2 h/d *v.* ≥2 h/d in accordance with the Australian Sedentary Behaviour Guidelines, which recommend that adolescents aged 12–18 years should not spend more than 2 h/d participating in screen time for recreational purposes^(^ ^23^ ^)^. The test–retest reliability of these and other survey items was tested in a separate sample of eighty-three Year 11 students with a 2-week interval between administrations (15·8d on average). Moderate to substantial test–retest reliability was observed for these three dichotomous variables (79 to 99 % agreement). Playing e-games was dichotomized as ‘does not play e-games’ and ‘plays e-games’ (86 % test–retest agreement) as a large proportion of participants did not play e-games for more than 2 h/d.

Total time spent sitting on a usual weekday and a usual weekend day, excluding travel in a motor vehicle, was self-reported as separate items using items from the International Physical Activity Questionnaire – Long Form^(^ ^24^ ^)^. Responses for weekdays were multiplied by 5 and usual weekend day multiplied by 2, then summed and divided by 7 to calculate average hours per day over a usual week^(^ ^25^ ^)^. Test–retest reliability of duration of sitting (h/d) was moderate (two-way mixed-effects model, absolute agreement, individual measure: intra-class correlation=0·53, 95 % CI 0·35, 0·67; *n* 80). Time spent sitting was treated as a continuous variable in all analyses.

#### Dietary intake

Participants reported the number of servings of fruit and the number of servings of vegetables they usually ate per day^(^ ^26^ ^)^. Fruit was defined as any fresh, dried, frozen or tinned fruit, excluding fruit juice. Vegetables were defined as any fresh, frozen or tinned vegetables, excluding potatoes, hot chips and fried potato. Response options were none, <1 serving, 1 serving, 2 servings, 3 servings, 4 servings, 5 servings and ≥6 servings daily. One serving of fruit was defined as equal to a medium piece or two small pieces of fruit, or one cup of diced pieces of fruit; one serving of vegetables was defined as equal to ½ cup of cooked vegetables or 1 cup of salad vegetables^(^ ^26^ ^)^.

Participants reported how much SSB and diet beverages, respectively, they usually drink (1 cup=250 ml). SSB included soft drinks, cordial, sport drinks and energy drinks. Diet beverages included the diet version of soft drinks, cordial and sport drinks. Response options for both items were none, <1 cup/week, 1–3 cups/week, 4–6 cups/week, 1–2 cups/d, 3–4 cups/d and ≥5 cups/d.

Consistent with definitions of energy-dense, nutrient-poor discretionary foods in the Australian Dietary Guidelines^(^ ^27^ ^)^, discretionary snacks were defined as consumption of dairy-based desserts, salty snacks from grains or starchy vegetables, sweet snacks such as sweet biscuits, cakes and muffins, and confectionery^(^ ^28^ ^)^. Discretionary snacks were represented by five items^(^ ^29^ ^)^. Participants reported how frequently they usually ate: (i) ice cream, icy poles and ice blocks; (ii) hot chips, wedges and fried potato; (iii) potato crisps and other salty snacks; (iv) confectionery such as lollies and chocolates; and (v) sweet biscuits, cakes and muffins. Response options (and coding) were: never (0), <1 time/week (0·07), 1–2 times/week (0·20), 3–4 times/week (0·50), 5–6 times/week (0·80), once a day/every day (1), 2 times/d (2) and ≥3 times/d (3). These items were summed. An additional item, usual frequency of fast-food intake, including meals or snacks from any takeaway food place, was reported on the same scale.

As the majority of dietary variables were negatively skewed, all were dichotomized in accordance with the Australian Dietary Guidelines^(^ ^27^ ^)^. Servings of fruit was dichotomized as ≥2 servings/d *v.* <2 servings/d. Initially, servings of vegetables was dichotomized as ≥5 servings/d *v.* <5 servings/d; however, very few participants consumed ≥5 servings/d. As such, servings of vegetables was dichotomized as ≥3 servings/d *v.* <3 servings/d. The dietary guidelines recommend that SSB, fast foods and discretionary snacks be consumed only occasionally and in small amounts; therefore regular SSB and diet beverages were dichotomized as <1 cup/week *v.* ≥1 cup/week, fast-food intake as <1 time/week *v.* ≥1 times/week, and discretionary snacks as <1 time/d *v.* ≥1 times/d. Test–retest reliability was good to excellent (71−95 % agreement) for these six dichotomous variables.

#### Covariates

Age, sex, BMI *Z*-score, participant’s residential location (rural *v.* urban), recruitment method (via school or social media), and mother’s and father’s highest education level were examined as potential covariates. Self-reported height and weight were used to calculate participants’ BMI *Z*-score using the age- and sex-specific BMI percentiles based on the WHO’s BMI-for-age cut-offs^(^ ^30^ ^)^. Rural and urban location of the participants’ residence was defined based on the Australian Bureau of Statistics’ Remoteness Index^(^ ^21^ ^)^. Both the mother’s and father’s highest education were reported by the participant with seven response options: (i) never attended school; (ii) primary school; (iii) some high school; (iv) completed high school; (v) technical or trades school certificate or an apprenticeship; (vi) university or tertiary qualification; and (vii) not applicable/no mother/father carer. For the analyses, the response options were combined into three categories: (i) some high school or less; (ii) completed high school, or technical or trades school certificate or an apprenticeship; and (iii) university or tertiary qualification^(^ ^31^ ^)^.

### Statistical analyses

All analyses were conducted using the statistical software package STATA/SE 14.0 (2015). Descriptive statistics were used to describe the sample characteristics and sedentary behaviour and dietary intake variables. Differences in these variables according to sex were determined using either *χ* ^2^ analyses or independent *t* tests. Regression was carried out to examine whether potential covariates (age, sex, BMI *Z*-score, participant’s residential location, recruitment method, and mother’s and father’s education) were associated with the predictor and outcome variables. Sex, residential location, and mother’s and father’s education were significantly associated with both sedentary behaviours and dietary intakes, except for paternal education; subsequent fully adjusted analyses adjusted for these covariates. Method of recruitment (school or social media) was associated with computer use and thus adjusted for in the analyses involving individual screen-based behaviours.

A series of crude and fully adjusted multiple logistic regression models were carried out to identify associations between: (i) each individual sedentary behaviour (e.g. TV viewing, computer use and e-games, entered into the same model) and each dietary variable; (ii) total screen time and each dietary variable; and (iii) total sitting time and each of the dietary variables. All analyses accounted for clustering by school. Fully adjusted analyses were repeated to include an interaction term between the sedentary behaviour variables and sex. Any significant sex interactions (indicated by a reduction in Akaike information criterion of >2 points^(^ ^32^ ^)^) were probed by repeating the models stratified by sex. Significance was set at *P* < 0·05. Only participants with complete data on each outcome (diet), predictor variable and covariate were included in the analytical sample (*n* 939; eighty-two exclusions).

## Results

Sample characteristics are presented in Table 1. The mean age of participants was 16·8 years and the sample comprised more girls than boys. Overall, 69 % of the sample spent ≥2 h/d in total screen time, just over a third spent ≥2 h/d using a computer for recreation, about one in four watched TV for ≥2 h/d and a similar proportion (overall) reported usually playing e-games. Comparatively few girls reported playing e-games compared with boys. Most participants met recommendations for fruit, 45 % consumed ≥3 servings of vegetables daily, a third drank at least one cup of SSB per week and a further 17 % consumed diet beverages. The proportion who reported consuming discretionary snacks at least daily was very high (70 %) and about a third reported eating fast foods on a weekly basis. There were no significant differences in demographic characteristics and sedentary behaviour variables between those included in the analyses compared with those excluded due to incomplete data. However, fewer of those included in the analyses consumed ≥1 cup of diet beverages per week (17 % *v.* 28 %, *P*=0·02) and fast foods at least once weekly (32 % *v.* 43 %, *P*=0·03).

**Table 1:** Characteristics of the sample of adolescents in School Year 11, Victoria, Australia, August 2013–June 2015; ProjectADAPT study

	Total (*n* 939)†	Boys (*n* 241)†	Girls (*n* 698)†
Characteristic	%	%	%
Age (years)			
Mean	16·9	16·9	16·8
sd	0·4	0·4	0·4
Overweight	12·9	13·9	12·5
Obese	4·2	2·5	4·8
Resident location			
Rural	30·1	33·6	28·9
Urban	69·9	66·4	71·1
Recruitment channel			
School	37·1	42·3	35·2
Facebook	62·9	57·7	64·8*
Mother’s highest education			
Some high school	16·9	15·4	17·5
Completed high school/tech/trade	25·0	24·5	25·2
University or tertiary qualification	58·0	60·2	57·3
Father’s highest education			
Some high school	17·0	12·8	18·4
Completed high school/tech/trade	34·5	32·7	35·2
University/tertiary qualification	48·5	54·4	46·4
Sedentary behaviours			
TV viewing, ≥2 h/d	25·8	23·2	26·7
Computer use, ≥2 h/d	36·2	37·3	35·8
E-games, no/yes	23·6	53·9	13·2***
Total screen time, ≥2 h/d	69·4	77·2	66·8**
Total sitting time (h/d)			
Mean	7·9	7·5	8·1
SD	2·7	2·9	2·7**
Dietary intakes			
Fruit, ≥2 servings/d	68·2	61·8	70·3*
Vegetables, ≥3 servings/d	45·1	40·3	46·7
SSB, ≥1 cup/week	33·3	51·0	27·2***
Diet beverages, ≥1 cup/week	17·4	22·4	15·6*
Discretionary snacks, ≥1 times/d	70·3	75·5	68·5*
Fast foods, ≥1 times/week	31·7	36·9	29·9*

TV, television; e-games, electronic games; SSB, sugar-sweetened beverages.

* *P* < 0·05; **P < 0·01; ***P < 0·001.

† There were additional missing data for weight status (total *n* 899; boys *n* 237; girls *n* 662).

### Individual screen-based sedentary behaviours and dietary intakes

Participants who watched TV for ≥2 h/d had 50 % and 68 % higher odds of consuming regular SSB and diet beverages, respectively, and 44 % higher odds of consuming discretionary snacks, than those who spent <2 h watching TV daily, independent of computer use and e-games. Those who used the computer for ≥2 h/d had 46 % and 39 % lower odds of consuming ≥2 servings fruit/d and ≥3 servings vegetables/d, respectively, and 50 % higher odds of consuming fast foods on more than one occasion each week, than those with computer time of <2 h/d, independent of time spent watching TV and using e-games. Participants who used e-games had 42 % lower odds of consuming ≥3 servings vegetables/d and 62 % higher odds of consuming regular SSB more than once per week compared with those who did not play e-games, independent of time watching TV and using a computer. There were no interactions by sex for any of the individual sedentary behaviours (Tables 2–4).

**Table 2:** Associations between sedentary behaviours and fruit and vegetable intakes in the sample of adolescents (*n* 939) in School Year 11 (mean age 16·8 years), Victoria, Australia, August 2013–June 2015; ProjectADAPT study

	Fruit, ≥2 servings/d				Vegetables, ≥3 servings/d			
	Crude		Fully adjusted		Crude		Fully adjusted	
	OR†	95 % CI	OR†	95 % CI	OR†	95 % CI	OR†	95 % CI
Individual behaviours‡								
TV ≥2 h/d (ref.: <2 h/d)	0·93	0·67, 1·27	0·93	0·67, 1·28	1·04	0·77, 1·40	1·06	0·79, 1·44
PC ≥2 h/d (ref.: <2 h/d)	0·54	0·39, 0·74*	0·54	0·38, 0·75*	0·58	0·44, 0·78*	0·61	0·46, 0·81*
Plays e-games (ref.: no)	0·63	0·45, 0·87*	0·74	0·51, 1·08	0·54	0·37, 0·79*	0·58	0·39, 0·86*
Total screen time§								
≥2 h/d (ref.: <2 h/d)	0·50	0·36, 0·68*	0·53	0·38, 0·73*	0·61	0·47, 0·79*	0·66	0·50, 0·86*
Total sitting time§								
Total sitting time (h/d)	0·93	0·88, 0·98*	0·92	0·87, 0·97*	0·94	0·89, 0·98*	0·93	0·89, 0·98*

TV, television; ref., reference category; PC, personal computer. For all analyses, significance is denoted by *P < 0·05.

† Crude OR: unadjusted for covariates. Fully adjusted OR: model including individual behaviours adjusted for sex, participant’s residential location and mother’s education.

‡ Logistic regression with TV, PC and e-games entered into the same model, accounts for clustering by school. Fully adjusted OR also adjusted for recruitment method.

§ Logistic regression, accounts for clustering by school.

**Table 3:** Associations between sedentary behaviours and beverage intakes in the sample of adolescents (*n* 939) in School Year 11 (mean age 16·8 years), Victoria, Australia, August 2013–June 2015; ProjectADAPT study

	Regular SSB, ≥1 cup/week				Diet beverages, ≥1 cup/week			
	Crude		Fully adjusted		Crude		Fully adjusted	
	OR†	95 % CI	OR†	95 % CI	OR†	95 % CI	OR†	95 % CI
Individual behaviours‡								
TV ≥2 h/d (ref.: <2 h/d)	1·45	1·06, 2·00*	1·50	1·09, 2·07*	1·72	1·21, 2·46*	1·68	1·17, 2·40*
PC ≥2 h/d (ref.: <2 h/d)	0·85	0·62, 1·18	0·85	0·61, 1·18	1·16	0·82, 1·64	1·19	0·85, 1·67
Plays e-games (ref.: no)	2·44	1·83, 3·26*	1·62	1·16, 2·26*	1·49	1·03, 2·17*	1·20	0·82, 1·77
Total screen time§								
≥2 h/d (ref.: <2 h/d)	1·67	1·25, 2·22*	1·48	1·11, 1·98*	1·44	0·97, 2·12	1·32	0·88, 1·98
Total sitting time§								
Total sitting time (h/d)	0·94	0·89, 0·99*	0·96	0·91, 1·01	0·97	0·91, 1·04	0·99	0·92, 1·06

SSB, sugar-sweetened beverages; TV, television; ref., reference category; PC, personal computer. For all analyses, significance is denoted by *P < 0·05.

† Crude OR: unadjusted for covariates. Fully adjusted OR: model including individual behaviours adjusted for sex, participant’s residential location and mother’s education.

‡ Logistic regression with TV, PC and e-games entered into the same model, accounts for clustering by school. Fully adjusted OR also adjusted for recruitment method.

§ Logistic regression, accounts for clustering by school.

**Table 4:** Associations between sedentary behaviours and snack and fast-food intakes in the sample of adolescents (*n* 939) in School Year 11 (mean age 16·8 years), Victoria, Australia, August 2013–June 2015; ProjectADAPT study

	Snacks, ≥1 times/d				Fast foods, ≥1 times/week			
	Crude		Fully adjusted		Crude		Fully adjusted	
	OR†	95 % CI	OR†	95 % CI	OR†	95 % CI	OR†	95 % CI
Individual behaviours‡								
TV ≥2 h/d (ref.: <2 h/d)	1·39	1·01, 1·92*	1·44	1·05, 1·98*	1·10	0·76, 1·58	1·09	0·75, 1·57
PC ≥2 h/d (ref.: <2 h/d)	1·04	0·76, 1·42	1·04	0·76, 1·42	1·56	1·18, 2·08*	1·50	1·13, 2·00*
Plays e-games (ref.: no)	1·44	0·98, 2·12	1·31	0·86, 1·98	1·43	1·04, 1·97*	1·21	0·84, 1·75
Total screen time§								
≥2 h/d (ref.: <2 h/d)	1·51	1·13, 2·03	1·50	1·12, 2·00*	1·95	1·44, 2·65*	1·80	1·33, 2·44*
Total sitting time§								
Total sitting time (h/d)	1·01	0·96, 1·07	1·02	0·97, 1·08	1·00	0·95, 1·05	1·00	0·95, 1·06

TV, television; ref., reference category; PC, personal computer. For all analyses, significance is denoted by *P < 0·05.

† Crude OR: unadjusted for covariates. Fully adjusted OR: model including individual behaviours adjusted for sex, participant’s residential location and mother’s education.

‡ Logistic regression with TV, PC and e-games entered into the same model, accounts for clustering by school. Fully adjusted OR also adjusted for recruitment method.

§ Logistic regression, accounts for clustering by school.

### Total screen time and dietary intakes

Participants who engaged in total screen time for ≥2 h/d had 47 % and 34 % lower odds of consuming ≥2 servings fruit/d and ≥3 servings vegetables/d, respectively, than those who spent <2 h/d engaged in screen time (Table 2). Participants who engaged in screen time for ≥2 h/d also had higher odds of consuming SSB (Table 3), discretionary snacks and fast foods (Table 4) than those who spent <2 h engaged in screen time daily. There were no significant interactions by sex.

### Total sitting time and dietary intakes

For every hour of time spent sitting, participants had 8 % and 7 % lower odds of consuming ≥2 servings fruit/d and ≥3 servings vegetables/d, respectively (Table 2). An interaction by sex was observed between total sitting time and diet beverages (*P*=0·05). The association was negative among males (OR=0·89; 95 % CI 0·78, 1·02, *P*=0·10) and positive among females (OR=1·03; 95 % CI 0·96, 1·12, *P*=0·353), but neither association was significantly significant.

## Discussion

The present study is one of the first to examine associations among a range of sedentary behaviours and healthy and unhealthy dietary intakes among adolescents. The findings suggest that screen-based sedentary behaviours, such as watching TV, using a computer and playing e-games (individually and in aggregate), are more strongly associated with unhealthy dietary intakes than overall sitting time. Of note, individual screen-based behaviours were associated with different dietary intakes. Using a computer and playing e-games were associated with fewer servings of fruits and vegetables, more frequent fast-food consumption and higher SSB intake, whereas TV viewing was associated with a higher consumption of both SSB and diet beverages and more frequent consumption of discretionary snacks. In contrast, total sitting time was only associated with lower consumption of fruits and vegetables. The lack of findings for total sitting time across other dietary variables examined in the present study may reflect the varied contexts in which sitting occurs (e.g. at school, on public transport).

The current finding that more than 2 h of TV viewing daily is associated with higher consumption of SSB, diet beverages and discretionary snacks is consistent with previous literature^(^ ^14^ ^,^ ^33^ ^)^. Studies have found that TV viewing is positively associated with fast-food intake and inversely associated with fruit and vegetable intake^(^ ^8^ ^,^ ^14^ ^)^. This could be due to a number of factors, including ‘mindless eating’ where a lack of attention to the quality and quantity of food consumed can lead to overeating^(^ ^34^ ^)^, advertising of SSB and discretionary snacks during peak viewing times of children^(^ ^35^ ^)^, or the sponsorship of fast foods and SSB on popular adolescents’ TV programmes and televised sport^(^ ^36^ ^)^. Family and peers may also influence both screen time and dietary behaviour among adolescents, although further research regarding peer influences on sedentary behaviours is required^(^ ^37^ ^)^. As most recreational screen time occurs in the home and much of the food adolescents eat is provided by families, supportive home environments that discourage excessive sitting and encourage healthy eating are critical.

A study of Brazilian adolescents found that behaviours such as eating meals in front of the TV and snacking while using screens are prevalent in the adolescent age group^(^ ^38^ ^)^. These habits may be linked to family practices that develop from an early age. Among children, the frequency of eating meals in front of the TV is inversely associated with healthy eating habits^(^ ^39^ ^,^ ^40^ ^)^ and positively associated with higher odds of being overweight or obese^(^ ^41^ ^)^. During childhood, the practice of eating meals in front of the TV, and the type of foods eaten, are largely regulated by other family members, and there is some evidence that ‘clustering’ of TV viewing and energy-dense food consumption could track from late childhood to adolescence and is associated with higher odds of being overweight or obese longitudinally^(^ ^42^ ^)^. Promoting healthy meal practices from an early age, such as not eating in front of the TV, may be important for creating healthy lifestyle habits later in adulthood.

While most studies have focused solely on the role of TV viewing, the present study found that total screen time was associated with consumption of fewer servings of fruits and vegetables, higher consumption of regular SSB and more frequent consumption of snacks and fast foods, and that this was not driven solely by TV viewing. Further, recreational computer use and playing e-games were also associated with similar unhealthy dietary intakes. These findings are consistent with other studies among children^(^ ^13^ ^,^ ^43^ ^)^ and together suggest that recreational screen use could be a useful indicator of poor dietary habits. The results suggest that, in addition to TV viewing, interventions should also focus on reducing other types of screen behaviours.

Although other studies examining TV viewing have reported inverse associations between TV viewing and fruit and vegetable intake, ours is one of the first studies in adolescents to report that overall screen time and total sitting time also have a negative impact on fruit and vegetable consumption. For example, the current study found those who engaged in screen time for ≥2 h/d had 34–47 % lower odds of consuming the recommended amount of fruits and vegetables, and for every hour spent sitting, the odds of consuming the recommended amount of fruits and vegetables was almost 10 % lower. This link could be partially explained by clustering of unhealthy behaviours, where unhealthy behaviours such as high sedentary time, unhealthy nutrition and insufficient physical exercise coexist^(^ ^44^ ^)^ and may share the same determinants.

The present study is one of few to examine associations between a range of sedentary behaviours and dietary habits among adolescents, which is a time of high risk for unhealthy lifestyles but also an opportune time to promote health. The findings provide support for government recommendations to limit screen time and prolonged sitting^(^ ^23^ ^)^ and suggest that it is possible that interventions designed to limit sedentary behaviour could have a beneficial impact on diet. The findings suggest that interventions that target both a reduction in individual and overall recreational screen time, as well as overall sedentary time, could contribute to healthier dietary intakes. Ensuring that evaluations of such interventions include measures of dietary intake will enable this possibility to be explored. However, the limitations of the current study must be acknowledged. The analyses were cross-sectional and all measures relied on self-report. It is therefore not possible to tease apart whether sedentary behaviour influences diet or vice versa, and while the items included were reliable, participant responses may have been influenced by social desirability bias. In addition, dietary intake while engaging in each of the specific screen-based sedentary behaviours was not specifically assessed. Further, the sample comprised a lower proportion of adolescents classified as overweight or obese (albeit height and weight were self-reported in the current study) and a higher proportion meeting guidelines for fruit and screen time, compared with national estimates for this age group^(^ ^15^ ^,^ ^45^ ^)^. The results may therefore not be generalizable to the wider population. Future studies should include longitudinal and experimental designs, examine a wider range of screen-based behaviours (laptops, tablets, smart phones), and consider collecting sedentary behaviour and dietary intake concurrently and/or information about the context of eating occasions. New technologies that provide objective measures of both behaviour and context, such as wearable cameras^(^ ^46^ ^)^, may provide promising insights into the nature of associations between sedentary behaviours and their context and dietary intakes.

## Conclusion

The results of the current study suggest that individual and aggregated recreational screen use (TV viewing, computer use and playing e-games), and total sitting time, are associated with unhealthy dietary intakes. Future studies should employ longitudinal designs and could explore if interventions that target both a reduction in individual and overall recreational screen time, as well as overall sedentary time, lead to healthier dietary intakes.
